# Malignant Proliferating Trichilemmal Tumor Treated with Radical Radiotherapy: A Case Report and Literature Review

**DOI:** 10.7759/cureus.999

**Published:** 2017-01-26

**Authors:** Duncan Sutherland, Kathryn Roth, Edward Yu

**Affiliations:** 1 Medical Imaging, Schulich School of Medicine & Dentistry, Western University, London, Ontario, CA; 2 Deptartment of Otolaryngology, Head and Neck Surgery, Western University, London, Ontario, CA; 3 Department of Radiation Oncology, London Regional Cancer Program, Western University, London, Ontario, CA; 4 Schulich School of Medicine & Dentistry, Western University, London, Ontario, CA

**Keywords:** malignant proliferating trichilemmal tumor, radiotherapy, surgical management, outer root sheath tumor, cosmesis, non-invasive, pilar tumor, skin cancer, trichilemmal tumor, proliferating trichilemmal cyst

## Abstract

Reported here is the first case of a malignant proliferating trichilemmal tumor treated with radical radiotherapy. Complete clinical response was achieved, and this obviated the need for aggressive surgery. These tumors have a tendency to develop in older patients, and have a propensity for affecting women more than men. The standard of treatment is surgical excision with a margin of normal tissue. Given that not all patients are good surgical candidates, the role of different treatment modalities in the management of this tumor is discussed.

## Introduction

The proliferating trichilemmal tumor (PTT), also known as the proliferating pilar tumor (PPT), is a rare, benign, neoplasm first described by Jones [1]. It is derived from the outer root sheath of the hair follicle. PPTs usually arise in the setting of a pilar cyst with a clinically rapid increase in the size of a previously small, asymptomatic lesion.  In PTTs, the presence of trichilemmal keratinization and lack of a granular layer are the histologic hallmarks [2]. It is usually a solitary lesion, tends to occur in the fourth to eighth decades of life, and has a predilection for women. PTTs have been estimated to occur on the scalp in close to 90% of cases. The rate of local recurrence and regional lymph node metastasis has been estimated to be between 3.7 - 6.6% and 1.2 - 2.6%, respectively [3].

Malignant proliferating trichilemmal tumors (MPTT) exhibit cellular dysplasia and invasion into the surrounding connective tissue. Histologic features that would lead to a diagnosis of MPTT, as opposed to a benign PTT, include the presence of abnormal mitoses, high mitotic counts, cellular pleomorphism, cytologic and architectural atypia, necrosis, infiltrating margins and aneuploidy. In fact, the metastatic rate of MPTTs with concerning features may be as high as 25% [3].

Owing to the rarity of MPTTs, diagnostic consideration should also be given to the relatively rare trichilemmal carcinoma, as well as the common squamous cell carcinoma. The trichilemmal carcinoma is skin tumor with a predilection for older patients. This entity has a tendency to occur in sun-exposed areas, such as the scalp, forehead, or neck. Trichilemmal carcinoma may occur as a nodular, papular, or exophytic solitary lesion. Histopathologically, trichilemmal carcinoma is a clear cell, lobular proliferation, centered on pilosebaceous structures. Their benign neoplastic counterpart, first described by Headington and French in 1962 [4], is the trichilemmoma with differentiation toward outer root sheath, or pilosebaceous follicular epithelium. The clinical significance of the trichilemmoma is in its association with multiple hamartoma syndrome (Cowden syndrome).

A case of a malignant PTT on the temple of a 93-year-old man is reported, and a brief overview of PTTs is provided. Given the large size of the lesion and the advanced age of the patient, neoadjuvant radiotherapy was initially planned to shrink the tumor prior to definitive surgical excision. A complete response of the lesion to radiotherapy was observed. The case is of interest because radiotherapy obviated the need for surgical resection. Adjuvant radiotherapy has been reported in the use of PTTs and is considered when metastatic spread is likely [5-6]. Surgical resection remains the primary treatment modality for MPTTs that have not metastasized. However, given the advanced age of the population most impacted, consideration of neoadjuvant radiotherapy with the aim of decreasing tumor size, followed by surgical resection, is an attractive option, in particular, if it is in a functionally or cosmetically sensitive facial region.

## Case presentation

**Figure 1:**
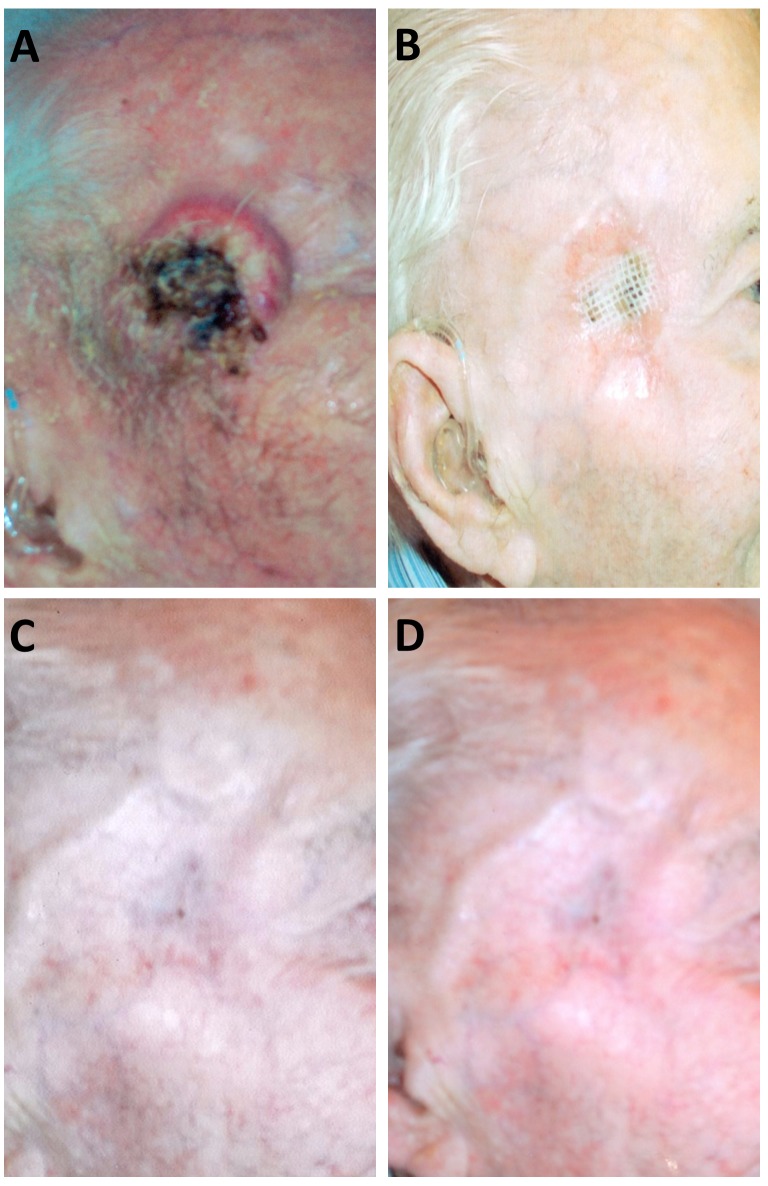
Clinical Photographs of Radical Radiotherapy Treatment of Malignant Proliferative Trichilemmal Tumor. A) Ulcerative lesion on initial presentation to clinic; B) Three months following radiotherapy treatment of tumor; C) Seven months following radiotherapy treatment of the tumor; D) Two years following treatment of the tumor. The patient was treated with 4,500 cGy divided over 15 doses (3 weeks) of 9 MeV electrons. Note the residual temple hollowing with resolution of MPTT lesion and radiotherapy skin changes. MPTT: malignant proliferating trichilemmal tumors

The patient was a 93-year-old man who presented with a several months history of an expanding lesion of the right temple (Figure 1). Medical history was significant for ischemic heart disease and elevated cholesterol. The clinical evaluation demonstrated the lesion was greater than 5 cm in greatest dimension, ulcerative, non-mobile, and extended superiorly into the temporal region and anteriorly overlying the zygoma region. The sebaceous material could be expressed with pressure. Clinically, he had no adenopathy. Punch biopsy pathology demonstrated the presence of a proliferating trichilemmal cyst in the dermis. In focal areas, marked atypia and invasion into the stroma was also noted. The findings were consistent with a malignant proliferative trichilemmal tumor. 

**Figure 2:**
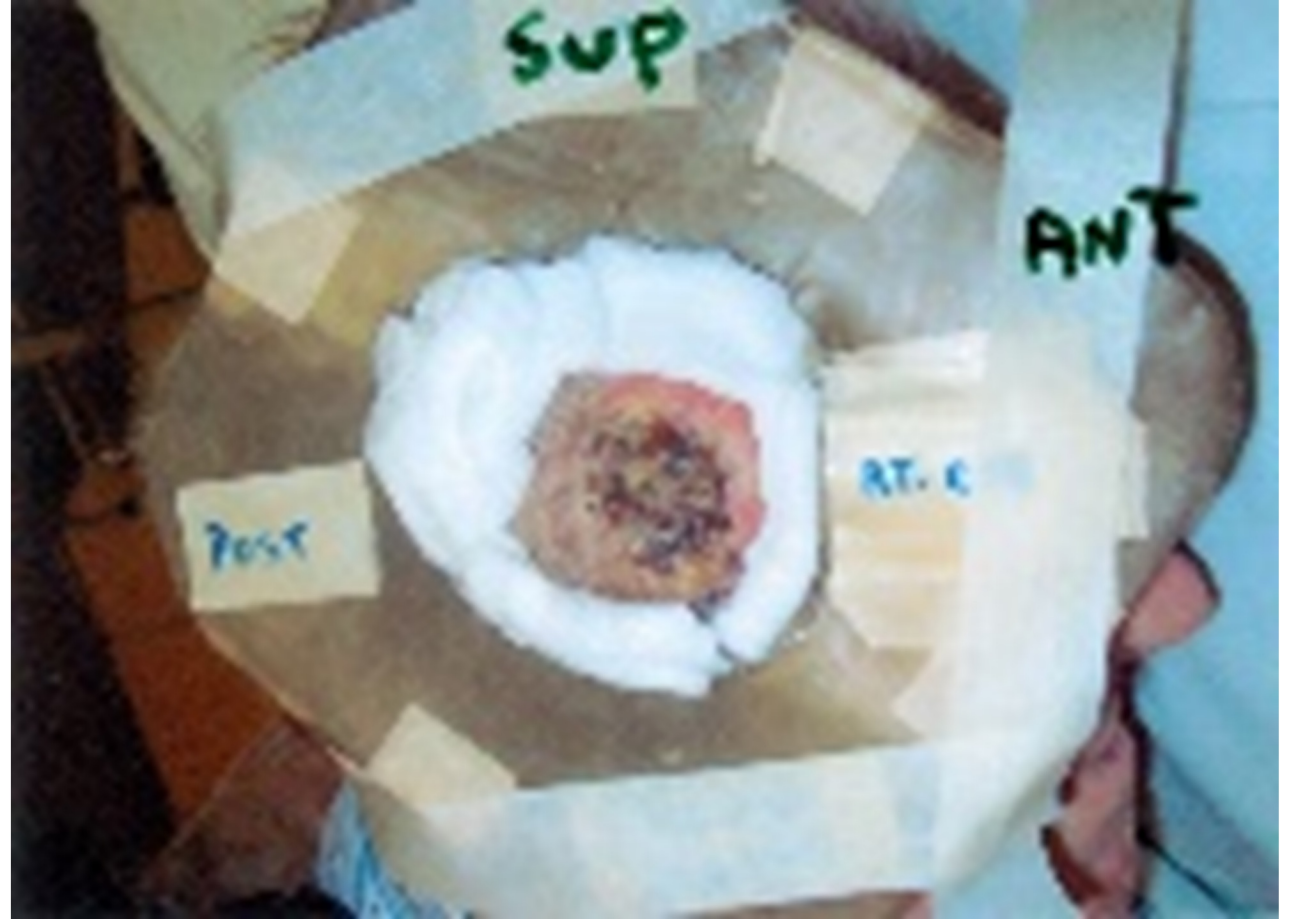
Customized Lead Shielding for Patient Treatment, Including Right External Eye Lead Shield. Note the superior (Sup), anterior (Ant), and posterior (Post) borders are demarcated in marker on the shielding.

The patient was reviewed at a multidisciplinary tumor board. Given the relatively large nature of this unusual tumor, an immediate surgical intervention was not felt to be in keeping with optimal management. Given his advanced age and medical comorbidities, he was deemed to be high-risk for a general anesthetic, and the broad, thick lesion with margins would have necessitated a larger resection with skin graft reconstruction. The location of the temporal branch of the facial nerve would have necessitated a limited deep margin resection. The patient was consented for radiotherapy totaling 4,500 cGy over three weeks of 9 MeV electrons with a margin of 2 cm. A bolus of 1.5 cm was used to ensure a 95 – 100% dose to the lesion. Customized lead shielding was used to minimize radiation to healthy tissue (Figure 2). Surgical reassessment and potential excision could then be arranged for a smaller resection, if needed.  

The patient was reassessed five weeks following radiotherapy and near complete response of the tumor was noted. Only a small area of crusting 1.5 cm from the lateral canthus was observed. This area was in keeping with radiation-induced changes to the tissue. Further healing was noted at six months with no evidence of a trichilemmal tumor. There was temple hollowing as a result of the loss of the fat pad. The patient continued to have no palpable parotid or neck adenopathy.

He was further seen by surgical oncology and they concurred with the assessment of complete clinical response. Given this response, surgery was not considered to be of further benefit. He was seen in follow-up for an additional two years and five months without recurrence. He died from an unrelated myocardial infarction. Prior to his death, written consent was obtained from the patient for this report.

## Discussion

MPTT is a rare neoplasm with fewer than 100 cases reported in the 50 years since it was first described [7]. Histologically, this malignancy may mimic squamous cell carcinoma (SCC) and was at one point thought to be a form of SCC arising from a sebaceous cyst. The distinguishing features that favor MPTT over SCC include trichilemmal-type keratinization, the lack of a granular layer of cells, and the absence of a premalignant epidermal lesion.

The pathologic sequence of events leading to the development of an MPTT is thought to start with trichilemmal cysts (TCs) as the original lesion. In the case of TCs, approximately 90% occur on the scalp. These lesions are smooth, round, and affect women more often than men. TCs may undergo neoplastic transformation to PTTs due to trauma, irritation, or chronic inflammation [8]. In this way, the heterogeneous nature of PTTs with both benign and malignant areas co-existing adjacent to each other are a natural consequence of inflammation in TCs. PTTs exhibit well-circumscribed borders without infiltration into adjacent tissue. They also exhibit broad bands of proliferating epithelial cells. Fungation of the overlying epithelium can occur in large, rapidly progressing, or longstanding lesions. In the final step of malignant development, complete loss of p53 has been implicated as one mechanism of the transformation of a PTT to an MPTT. MPTTs exhibit both invasion into surrounding tissue and severe cellular atypia.

The mainstay treatment of MPTT is surgical excision with a 1-cm margin of normal tissue [2]. However, given the relatively high local recurrence rate (3.7 - 6.6%) [3] with a median literature follow-up of patients for 15 months, other treatment modalities are being investigated, including Mohs surgery and excision with frozen section margin assessment [9]. These surgical techniques have the benefit of decreasing the rate of recurrence while simultaneously sparing tissue in larger lesions. To further limit the rate of recurrence, some groups have advocated for adjuvant radiotherapy in the treatment of MPTT [10] while palliative radiotherapy has also been used in metastatic disease. Cyclophosphamide/adriamycin/vincristine (CAV)-based chemotherapy has been shown to be somewhat effective. If metastatic disease is suspected, a potential work-up advocated by several groups includes the use of a CT scan with contrast of the head and neck for scalp lesions, chest x-ray, and whole body PET scan to rule out distant metastatic disease [2, 9].

## Conclusions

This case report demonstrates the management of a malignant proliferating trichilemmal tumor with radical radiotherapy. The patient was a nonagenarian and tolerated the procedure well. Surgical excision with a margin of normal tissue is standard treatment. However, given the large size of the lesion, advanced age of the patient, and the potential for surgical complications, such as facial nerve damage, radiotherapy was initially used to reduce tumor size. A complete clinical response of the tumor was observed, and no further treatment was deemed necessary. To our knowledge, this is the first report of such a tumor being treated with radical radiotherapy. Neoadjuvant radiotherapy has the potential to be an excellent add-on for surgical cases. It may also be a good alternative to surgery in elderly patients, frail patients, or in those with large tumors in either cosmetically or functionally significant areas.

## References

[1] Jones EW (1966). Proliferating epidermoid cysts. Arch Derm.

[2] Satyaprakash AK, Sheehan DJ, Sangueza OP (2007). Proliferating trichilemmal tumors: a review of the literature. Dermatol Surg.

[3] Ye J, Nappi O, Swanson PE, Patterson JW, Wick MR (2004). Proliferating pilar tumors: A clinicopathologic study of 76 cases with a proposal for definition of benign and malignant variants. Am J Clin Pathol.

[4] Headington JT, French AJ (1962). Primary neoplasms of the hair follicle. Histogenesis and classification. Arch Dermatol.

[5] Takenaka H, Kishimoto S, Shibagaki R, Nagata M, Noda Y, Yasuno H (1998). Recurrent malignant proliferating trichilemmal tumour: local management with ethanol injection. Br J Dermatol.

[6] Park BS, Yang SG, Cho KH (1997). Malignant proliferating trichilemmal tumor showing distant metastases. Am J Dermatopathol.

[7] Lu ZR, Imhagwe G (2016). Malignant proliferating trichilemmal tumour: A case report and literature review. Pathology.

[8] Shimizu Y, Sakita K, Arai E, Tsuchida T, Ogawa F, Ban S, Mitsuhashi T, HIrose T, Shimizu M (2005). Clinicopathologic features of epidermal cysts of the sole: Comparison with traditional epidermal cysts and trichilemmal cysts. J Cutan Pathol.

[9] Fieleke DR, Goldstein GD (2015). Malignant proliferating trichilemmal tumor treated with Mohs surgery: proposed protocol for diagnostic work-up and treatment. Dermatol Surg.

[10] Siddha M, Budrukkar A, Shet T, Deshpande M, Basu A, Patil N, Bhalavat R (2007). Malignant pilar tumor of the scalp: A case report and review of literature. J Cancer Res Ther.

